# Health Disparities in Ischaemic Heart Disease Mortality in Hungary From 1970 to 2010: An Age-Period-Cohort Analysis

**DOI:** 10.2188/jea.JE20140122

**Published:** 2015-06-05

**Authors:** Krisztina Gero, Ehab S. Eshak, Enbo Ma, Hideto Takahashi, Hiroyuki Noda, Hiroyasu Iso

**Affiliations:** 1 Public Health, Department of Social Medicine, Osaka University Graduate School of Medicine, Suita, Osaka, Japan; 1 大阪大学大学院医学系研究科社会医学講座公衆衛生学; 2 Department of Clinical Trial and Clinical Epidemiology, Faculty of Medicine, University of Tsukuba, Tsukuba, Ibaraki, Japan; 2 筑波大学医学医療系臨床試験・臨床疫学; 3 Cancer Control and Health Promotion Division, Health Service Bureau, Ministry of Health, Labour and Welfare, Tokyo, Japan; 3 厚生労働省健康局がん対策・健康増進課

**Keywords:** ischaemic heart disease, mortality, age-period-cohort, Hungary

## Abstract

**Background:**

The objective of this study was to examine long-term trends in rates of ischaemic heart disease (IHD) mortality, a leading cause of mortality in Hungary. The study examined the effects of age, period, and cohort on IHD mortality rates and compared mortality rates between the capital (Budapest) and non-capital counties.

**Methods:**

Data on IHD deaths and population censuses were obtained from the Hungarian Central Statistical Office. Age-period-cohort analysis utilized nine age-group classes for ages 40 to 84 years, eight time periods from 1970 to 2009, and 16 birth cohorts from 1886 to 1969.

**Results:**

Age-adjusted IHD mortality rates for men and for women generally increased from 1970 to 1993 and from 1980 to 1999, respectively, decreasing thereafter for both sexes. IHD mortality rates for men and for women from Budapest were lower from 1991 and from 1970, respectively, than corresponding rates in non-capital counties, with the difference increasing after 1999. Age had a more significant influence on mortality rates for women than for men. The period effect increased from 1972 to 1982 and decreased thereafter for men, while the period effect decreased consistently for women from 1972 to 2007. The decline in period effect for both sexes was larger for individuals from the capital than for those from non-capital counties. The cohort effect for both sexes declined from birth years 1890 to 1965, with a steeper decline for individuals from the capital than for those from non-capital counties.

**Conclusions:**

The findings indicate a need for programs in Hungary for IHD prevention, especially for non-capital counties.

## Abstract (ja)

**目的:**

本研究の目的は、ハンガリーにおける主要な死因のうち、虚血性心疾患（IHD）死亡率の長期的な傾向を検討することにある。本研究では、ハンガリー全土ならびに首都（ブダベスト）と他郡の比較について、IHD死亡率のAPC(age-period-cohort)分析を行った。

**方法:**

IHDによる死亡と人口センサスのデータは、ハンガリー中央統計局から得た。APC分析は、40~84歳を対象として5年齢ごとの9クラスとし、1970~2009年までの死亡を8期間に分けて、1886~1969年までの16コホートを構築した。

**結果:**

年齢調整IHD死亡率は、男性では1970年から1993年、女性では1980年から1990年までは上昇傾向であったが、その後は男女ともに減少した。さらに、男性では1991年、女性では1970年以降は、首都のIHD死亡率は他郡よりも低く、その差が1990年以降は増大した。年齢効果は、男性よりも女性のほうが大きく影響した。男性の時代効果は、1972年～1982年は増加し、その後は減少した一方で、女性は1972年～2007年の間は一貫して減少した。首都での時代効果の減少は、男女ともに他郡よりも低かった。男女ともにコホート効果は1890年～1965年の出生者で減少し、また、首都におけるコホート効果は、他郡よりも急峻に減少した。

**結論:**

ハンガリーにおいて、特に首都以外の郡におけるIHD防止のためのプログラムの必要性が示された。

## INTRODUCTION

Ischaemic heart disease (IHD) is one of the major causes of death in Hungary. In 1970, the rate of premature IHD mortality (before the age of 65) in Hungary was similar to that in the European Union (average based on mortality rates of member states joining prior to 2004) for men but higher than that in the European Union for women.^1^^,^^2^ The rates of premature IHD mortality increased for both men and women in Hungary between 1970 and the mid-1990s, while that in western European countries has substantially declined since the 1970s.^1^^–^^7^ Therefore, the rate of premature IHD mortality in Hungary was about 3 times higher in 1995 and 4 (for men) to 5 (for women) times higher in 2010 than that in the European Union, surpassing the rates of most European countries, with the exception of some post-Soviet states.^1^^,^^2^ In addition, there were clear differences in lifestyles and prevalence of IHD risk factors between urban and rural populations in Hungary,^8^^–^^12^ potentially leading to differences in mortality rates between the capital and non-capital counties. However, no previous studies have compared IHD mortality rates between the capital and non-capital counties.

Age-period-cohort (APC) analysis can separately assess the effects of chronological age (age effects); factors taking effect around the time of death, risk factors affecting the entire population, and preventive measures or new medical procedures and treatments (period effects); and effects of risk factors and environmental exposures shared by each generation (cohort effects).^13^^–^^16^

In the current study, we examined long-term trends in IHD mortality rates for Hungarians by estimating the effects of three major time-dependent components of mortality trends: age, cohort (birth year), and period (calendar year or death year). We separately analysed the data for the capital and non-capital counties and compared trends in IHD mortality, as well as age, period, and cohort effects, to gain a better understanding of factors influencing IHD trends and their geographical differences.

## MATERIALS AND METHODS

### Population data and age-standardised mortality rates

The Hungarian Central Statistical Office provided the data on the number of deaths and mid-year population by gender, age group, and place of residence. For confidentiality reasons, data were undisclosed if the number of deaths equalled one or two in a certain age group. As figures from missing data equalled either one or two, calculations used the value 1.5.

Since 1870, the population census survey has been conducted once every 10 years in Hungary. The population aged 40 to 84 years has gradually grown from 1.95 million in 1970 to 2.20 million in 2011 for men and from 2.32 million to 2.72 million for women, respectively.^17^^,^^18^ The corresponding population growth in the capital was from 0.40 million to 0.36 million for men and from 0.52 million to 0.48 million for women, and corresponding growth in non-capital counties was from 1.56 million to 1.84 million for men and from 1.80 million to 2.24 million for women.^17^^,^^18^ From 1970 to 1990, 21%–22% of the population aged 40 to 84 years lived in the capital; however, this number decreased slightly to 18% in 2001 and to 17% in 2011. Between 1995 and 2011, the respective in- and out-migrations of the population aged 40 to 84 years in the capital were 1.5%–2.6% and 1.9%–3.1% for men and 1.3%–2.1% and 1.5%–2.3% for women.^12^

Three revisions of the International Classification of Diseases (ICD) were published between 1970 and 2010: the 8th revision (ICD-8), 9th revision (ICD-9), and 10th revision (ICD-10), which were published in 1970, 1979, and 1996, respectively. IHD was defined by codes 410–414 in ICD-8 and ICD-9, and by codes I20–I25 in ICD-10. All registered deaths from IHD in Hungary were included in the analysis. Autopsy or a physician determined the cause of death according to the European Union standards. Between 1995 and 2010, 26.2%–38.3% of the IHD cases were coded based on autopsy findings, and the rate of unspecified causes of death in the ICD XVIII sector was below 0.14%.^12^

We defined nine 5-year age classes for ages 40 to 84 years in the study. Mortality rates (number of deaths per population of 100 000 per year) for the entire country (20 counties, including the capital) were calculated, as were rates for the capital and non-capital counties (ie, the remaining 19 counties). We compared mortality rates for the entire country and the capital, as well as the combined mortality rates of the 19 non-capital counties. The direct method of age-standardisation, based on the Hungarian sex-specific population aged 40 to 84 years in 1990, was performed.

### APC analysis

To conduct the APC analysis, we divided the period of 1970 to 2009 into 5-year intervals denoted by the calendar year midway through each interval. Birth cohorts were obtained by using cells diagonally arranged from upper left to lower right in an age-by-period table. From 1886 to 1969, 16 birth cohorts of 5-year intervals were defined, with each cohort denoted by the year midway through the interval. We estimated person-years for each period using the corresponding mid-year population data.

The model used for APC analysis is described in detail elsewhere.^13^ In summary, the effects of age, cohort, and period were assessed separately using a Poisson regression model. This model included the participants’ ages, birth years, and calendar years of death from IHD as explanatory variables. Aliasing due to the linear dependence between these three factors can occur; to avoid this well-known problem, we used the non-linear (curvature) approach introduced by Holford.^19^ This concept divides the full effect of each age, period, and cohort into two parts: the ‘linear part’ and the ‘non-linear part’. The linear part roughly estimates the overall slope of each effect curve, and the non-linear part is the deviation of the original effect curve from this approximated line, which expresses the original change pattern of each effect. The non-linear part of each effect is calculated independently. In the APC model, the linear parts of the effects of age and period are estimated with the assumption that the linear cohort effect is zero, while the linear cohort effect is estimated with the assumption that the linear period effect is zero. The age group 40 to 44 years, the period 1970 to 1974, and the birth cohort of 1890 were used as reference. The goodness of fit of each effect model was determined by dividing the likelihood ratio G2 statistic by the difference in degrees of freedom from the full models.

Statistical analysis was performed using SAS version 9.3 (SAS Institute Inc., Cary, NC, USA) and R version 2.15.1 for Windows (R Foundation for Statistical Computing, Vienna, Austria).

## RESULTS

### Secular trends in mortality

**Figure 1.**
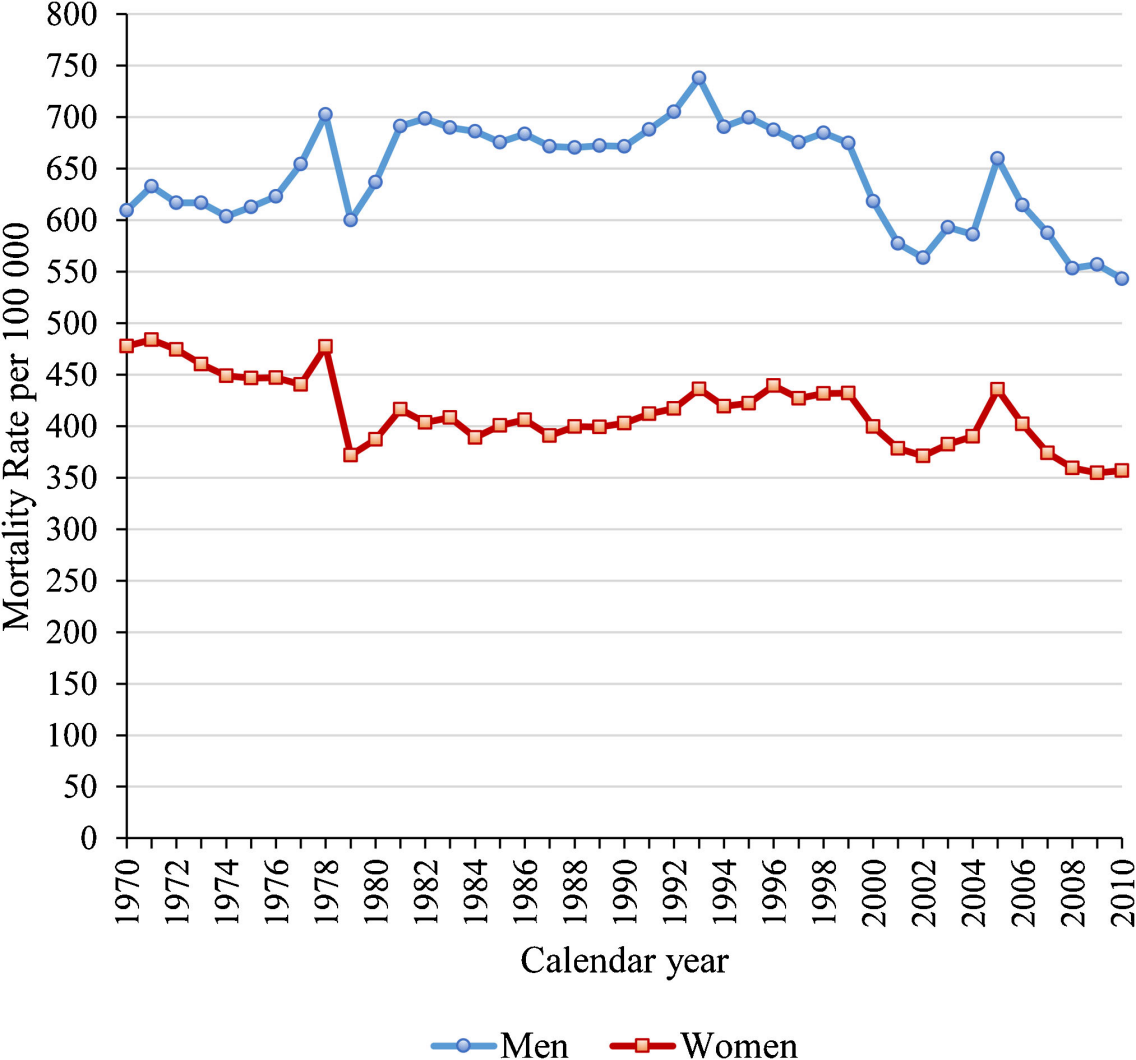
Trends in sex-specific, age-adjusted mortality rates of ischaemic heart disease for men and women aged 40–84 years in Hungary.

Table 1 and Figure 1 show sex- and age-specific and age-standardised mortality rates for IHD in Hungary between 1970 and 2010. The IHD mortality rate per 100 000 population for men increased from 609.5 in 1970 to 737.9 in 1993 and decreased thereafter, reaching 543.3 in 2010. The corresponding rate for women decreased from 477.8 in 1970 to 440.5 in 1977, increased between 1980 and 1999 from 387.3 to 432.0, and then decreased to 357.0 in 2010. Rates for both sexes fluctuated during 1979 and 2005. Throughout the periods examined, IHD mortality rates were 22%–43% lower for women than for men.

**Table 1.** Sex- and age-specific mortality rates^a^ (per 100 000) of ischaemic heart disease in Hungary from 1970 to 2010

Age (years)	1970	1975	1980	1985	1990	1995	2000	2005	2010
Men									
40–44	71.9 (257)	79.6 (269.5)	126.1 (398)	119.4 (415)	104.0 (378)	120.0 (495)	86.0 (307)	74.7 (225.5)	54.4 (188)
45–49	112.2 (391)	157.1 (546)	191.7 (626)	200.6 (607)	197.1 (643)	203.2 (701)	152.8 (600)	153.0 (531)	113.6 (334)
50–54	197.3 (418)	219.6 (734)	333.9 (1100)	347.2 (1065)	314.1 (877)	310.7 (939)	255.3 (820)	266.6 (995)	216.2 (710)
55–59	322.9 (965)	336.4 (670)	482.8 (1497)	538.8 (1616)	533.4 (1470)	477.7 (1196)	388.6 (1063)	405.8 (1217)	345.1 (1186)
60–64	563.4 (1509)	585.9 (1585)	671.5 (1201)	728.0 (1978)	747.7 (1935)	759.2 (1804)	649.4 (1410)	598.7 (1501)	519.0 (1392)
65–69	992.1 (2108)	907.0 (2064)	962.3 (2205)	1010.3 (1500)	1021.5 (2265)	1105.3 (2327)	972.8 (1903)	953.6 (1841)	753.7 (1626)
70–74	1498.0 (2222)	1424.6 (2318)	1401.6 (2451)	1548.0 (2667)	1435.2 (1613)	1620.0 (2700)	1471.0 (2347)	1496.2 (2439)	1248.3 (1946)
75–79	2333.0 (1864)	2315.7 (2261)	2077.4 (2195)	2095.4 (2356)	2172.3 (2446)	2197.6 (1672)	2150.0 (2415)	2509.1 (2975)	2050.0 (2434)
80–84	3762.5 (1458)	3802.0 (1592)	2834.6 (1452)	3077.0 (1654)	3179.0 (1863)	3516.0 (2166)	3267.3 (1425)	4184.1 (2962)	3521.5 (2587)
All^b^	609.5	612.7	637.0	675.6	671.6	699.7	618.4	660.0	543.3
Women									
40–44	13.5 (51)	14.6 (52)	20.3 (69)	25.9 (92)	26.6 (98)	23.1 (97.5)	23.1 (84.5)	19.7 (60.5)	12.9 (44)
45–49	25.1 (97)	31.0 (115)	46.1 (161.5)	52.8 (176.5)	47.8 (164.5)	49.2 (178)	38.0 (157.5)	35.3 (130.5)	25.6 (78)
50–54	57.4 (139)	61.6 (232)	77.9 (282)	76.3 (260)	78.7 (253)	73.5 (246)	64.0 (225.5)	62.0 (256)	59.4 (215)
55–59	104.9 (362)	114.7 (268)	132.5 (484)	158.4 (551)	137.4 (445)	145.1 (448)	99.8 (322)	103.4 (361)	101.6 (407)
60–64	237.4 (751)	250.3 (817)	234.2 (520)	265.6 (912)	267.7 (869)	258.1 (787)	205.7 (601)	195.4 (625)	160.0 (536)
65–69	492.8 (1287)	453.2 (1308)	460.8 (1379)	432.0 (873)	462.5 (1427)	463.9 (1374)	431.5 (1208)	389.7 (1108)	293.9 (885)
70–74	948.9 (1969)	872.1 (1938)	788.8 (1951)	815.7 (2077)	786.7 (1348)	813.4 (2165)	789.9 (2041)	799.4 (2083)	624.2 (1606)
75–79	1800.1 (2344)	1684.8 (2621)	1301.3 (2185)	1355.6 (2544)	1380.2 (2662)	1386.8 (1875)	1481.3 (3122)	1632.4 (3598)	1304.6 (2855)
80–84	3220.1 (2171)	2874.8 (2275)	2240.3 (2176)	2283.2 (2381)	2304.8 (2696)	2689.8 (3398)	2501.4 (2302)	3154.2 (4883)	2701.5 (4335)
All^b^	477.8	446.7	387.3	400.9	402.8	422.4	399.5	435.6	357.0

^a^Rates are based on 5-year age groups for ages 40–84 years. The numbers of deaths are shown in parentheses.

^b^Mortality rates were standardised through the direct method, with the Hungarian population aged 40–84 years in 1990 used as standard.

**Figure 2.**
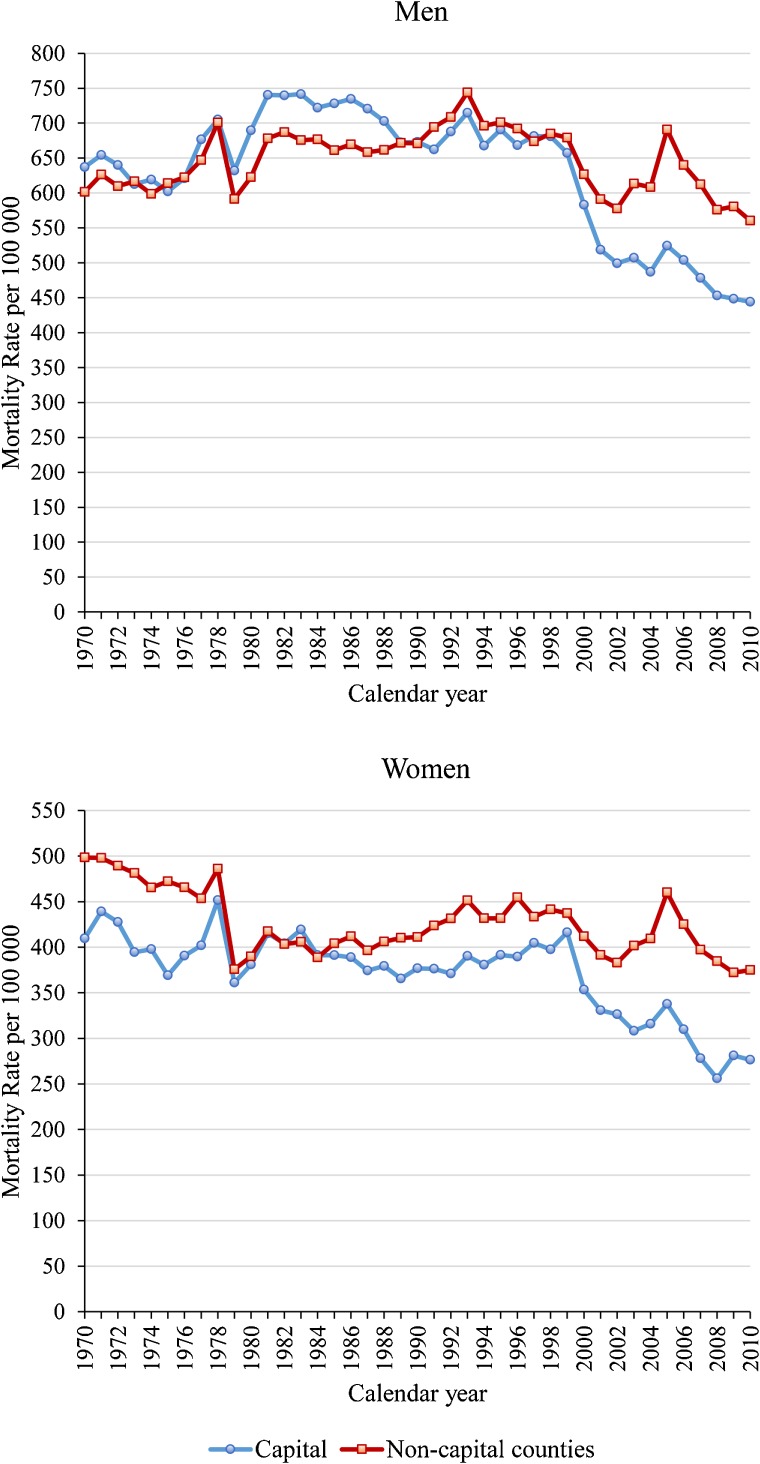
Trends in sex-specific, age-adjusted mortality rates of ischaemic heart disease for men and women aged 40–84 years from the capital and non-capital counties of Hungary.

Table 2 and Figure 2 show sex-specific trends in age-adjusted mortality rates for IHD in the capital and non-capital counties of Hungary. While the IHD mortality trends for both sexes in the capital and non-capital counties followed a pattern similar to that for the entire country, the rates for the capital after 1999 declined substantially.

**Table 2.** Sex-specific, age-adjusted mortality rates^a^ (per 100 000) of ischaemic heart disease for men and women aged 40–84 years from the capital and non-capital counties of Hungary

Year	1970	1975	1980	1985	1990	1995	2000	2005	2010
Men									
Capital	637.2	602.3	689.8	728.3	673.1	690.7	583.1	524.5	444.3
Non-capital counties	601.4	614.0	622.7	661.4	671.0	701.3	626.7	690.7	560.6
Women									
Capital	409.7	369.0	381.1	391.0	376.8	391.5	353.3	337.7	276.4
Non-capital counties	498.5	472.2	389.8	404.1	411.1	431.7	412.0	460.3	374.8

^a^Mortality rates were standardised through the direct method, with the Hungarian population aged 40–84 years in 1990 used as standard.

The IHD mortality rate per 100 000 population between 1970 and 1988 for men from the capital was higher than that for men from non-capital counties, with an average of 680 versus 643, respectively; however, this trend reversed after 1990. The IHD mortality rate per 100 000 population for women from the capital was an average of 371 and was generally lower than that for women from non-capital counties, where the average was 426. In 2010, the IHD mortality rates for men and for women from the capital were 20.8% and 26.2% lower, respectively, than the corresponding rates for individuals from non-capital counties.

### APC analysis

The present study compared the goodness of fit of four different Poisson models applied to age-specific mortality rates for men and women from the capital and non-capital counties of Hungary. Among the age-only, age-cohort, age-period, and APC models, the APC model had the smallest value of G2/Δdf (*P* > 0.05 in any area for either sex). Among the four Poisson models, the APC model was most acceptable and most closely fit the data for both sexes in all examined areas in Hungary (eFigures 1–3).

**Figure 3.**
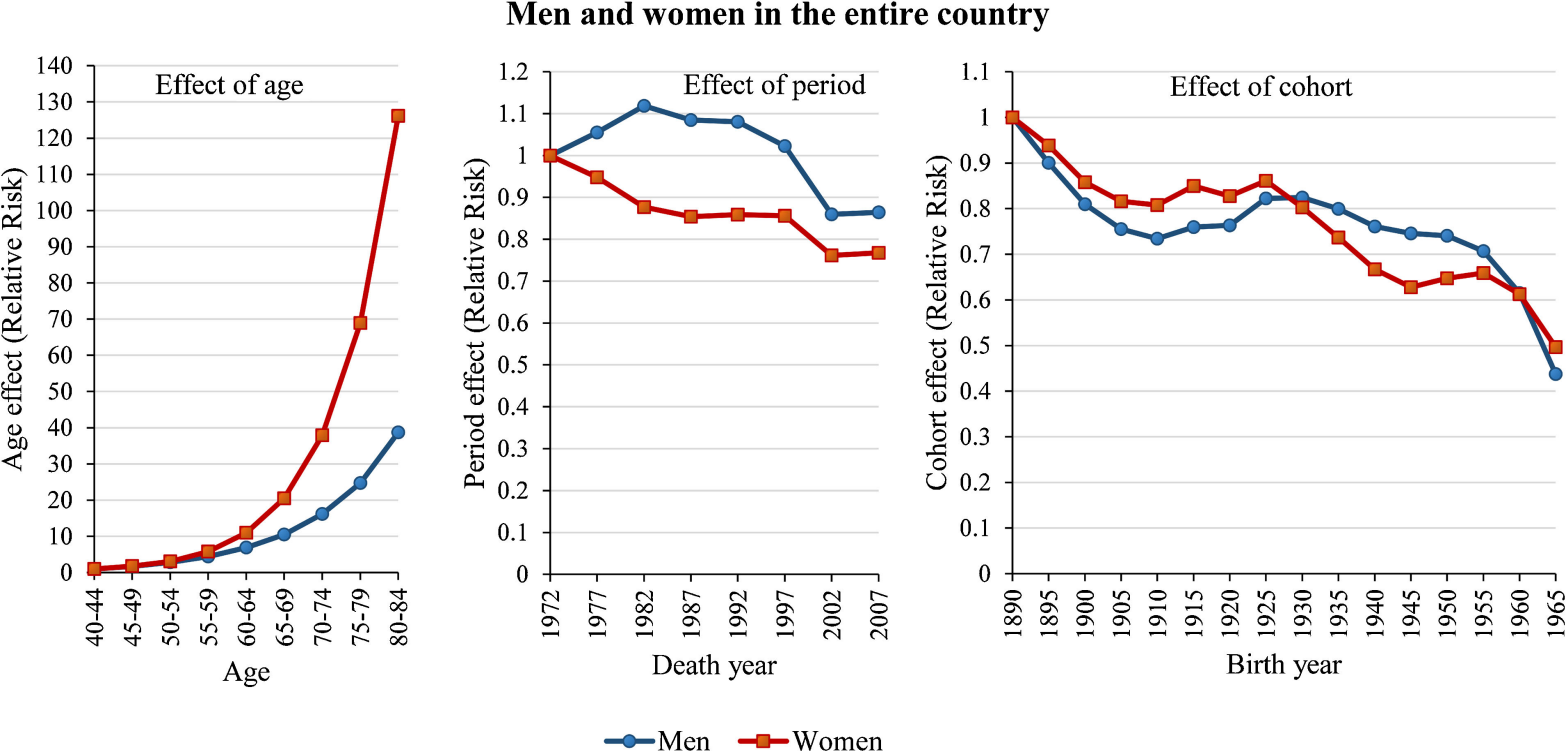
Effects of age, period, and birth cohort on ischaemic heart disease mortality for men and women aged 40–84 years in Hungary. *P*-values for the goodness-of-fit statistics of the APC model were 0.12 for men and 0.40 for women.

Figure 3 shows sex-specific effects of age, period, and cohort on IHD mortality in the entire country. The Y-axis of each plot represents the relative risk (RR) of mortality from IHD at the corresponding age, death year, or birth year of the deceased. The effect of age increased with increasing age for both sexes, reaching a RR 3.3 times larger for women than for men at ages of 80 to 84 years. The RR associated with the effect of period for men increased to 1.12 in 1982 and then decreased to 0.86 in 2002, after which it plateaued. The RR associated with the effect of period for women decreased to 0.76 in 2002 and plateaued thereafter. The RR associated with the effect of cohort on IHD mortality decreased to 0.44 for men and 0.50 for women in 1965, with temporary increases between 1910 and 1925 (from 0.73 to 0.82 for men and from 0.81 to 0.82 for women), and between 1945 and 1955 (from 0.63 to 0.66 for women).

**Figure 4.**
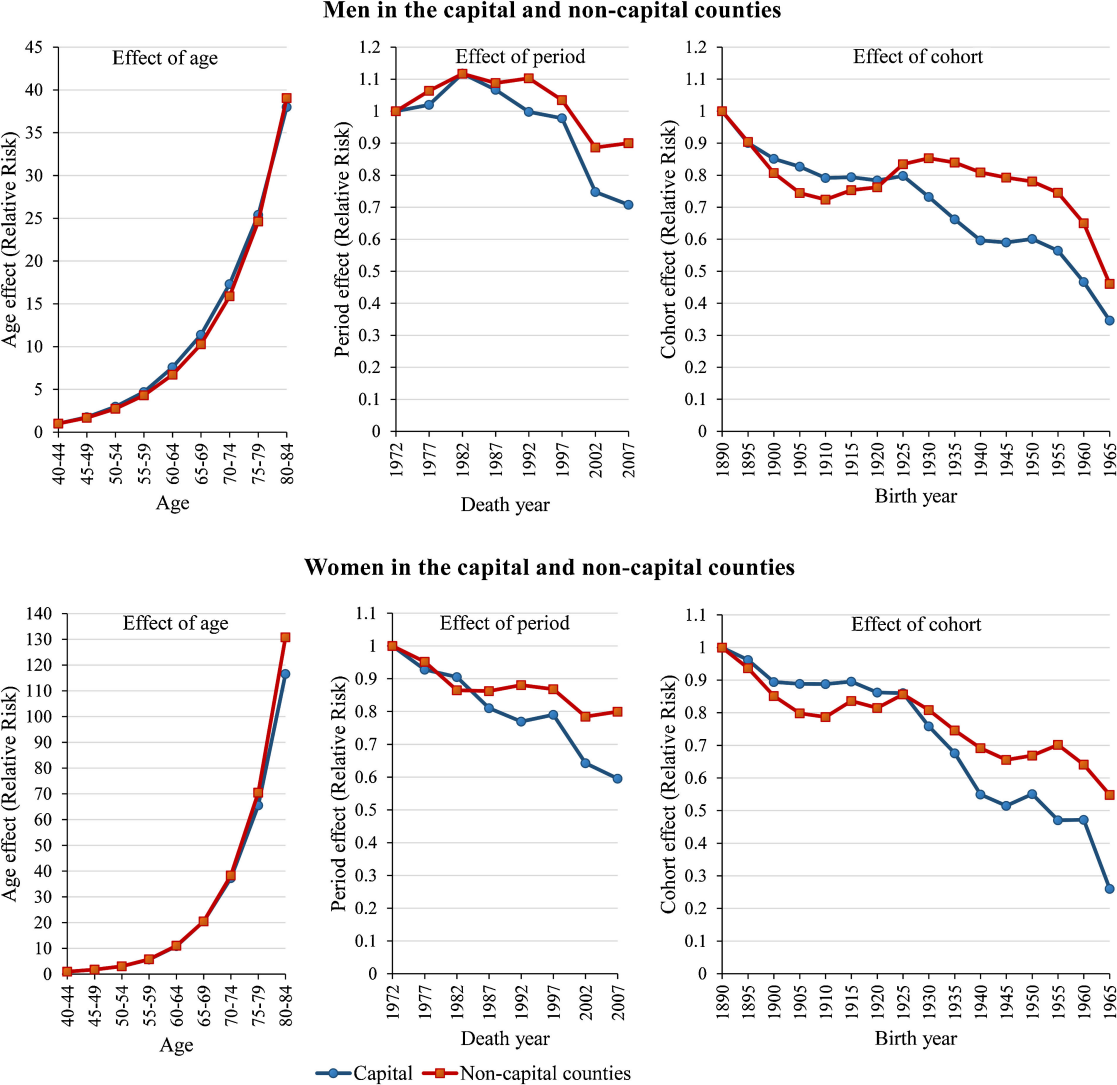
Effects of age, period, and birth cohort on ischaemic heart disease mortality for men and women aged 40–84 years in the capital and non-capital counties of Hungary. *P*-values for the goodness-of-fit statistics of the APC model were >0.999 for both sexes in the capital, and 0.43 for men and 0.78 for women in non-capital counties.

Figure 4 shows the results for the capital and non-capital counties. The effects of age on findings for both sexes from the capital and non-capital counties were similar. The RR associated with the effect of period for men from the capital and non-capital counties increased to 1.12 in 1982 and decreased thereafter, reaching 0.71 and 0.90, respectively, in 2007. The effect of period for women from the capital and non-capital counties decreased overall from 1972 to 2007, with the decline for women from the capital being steeper than that for women from non-capital counties. In 2007, the RR associated with the effect of period was 0.60 for women from the capital and 0.80 for women from non-capital counties. The effect of cohort for both sexes from the capital showed a greater decrease than that for individuals from non-capital counties. In 1965, the RR associated with the effect of cohort was 0.35 for men and 0.26 for women from the capital, while the respective numbers for men and women from non-capital counties were 0.46 and 0.55.

## DISCUSSION

We performed a long-term APC analysis for the period 1970 to 2010 and found that rates of IHD mortality increased for men until 1993 and increased for women until 1999, after an initial decrease between 1970 and 1977. The rates of IHD mortality decreased thereafter for both sexes. Additionally, we observed different trends in different parts of the country (ie, capital versus non-capital counties). Rates of IHD mortality in the capital decreased rapidly compared with those in non-capital counties, resulting in widened disparity in health for both sexes starting from the year 2000.

The increase in the number of cigarettes smoked per person per year and the increase in alcohol consumption between 1970 and 1995 correlated with the increase in IHD mortality rates for both sexes during the same period. In addition, the decrease in the percentage of regular daily smokers among men between 1995 and 2009 correlated with changes in IHD mortality rates during the same period (eFigures 4–6).^2^

Trends in alcohol intake (litres/capita) for the Hungarian population (eFigure 6)^2^—namely an increase between 1970 and 1980, a steady state between 1980 and 1990, and a decrease from 1990 to 1999—correlated with trends in IHD mortality rates for men. However, these correlations should be considered with caution because we did not have individual data on cigarette and alcohol consumption.

The increase in polyunsaturated fatty acid/saturated fatty acid ratio and the decrease in dietary cholesterol levels between 1992–94 and 2003–04 for both sexes corresponded with reduced rates of IHD mortality.^20^

After the end of communism in Hungary in 1989, the nation experienced temporary inflation, mass unemployment, and increasing disparity in socio-economic status. Approximately 52% of Hungarians reported deteriorating income and financial situations in 1994 compared with conditions 3 years prior, and 59% reported an unfavourable change in social status compared with the status in the 1980s.^11^ These changes might partly explain the accelerated rise in IHD mortality rates between 1989 and 1996.

The advancement of medical treatment for IHD, especially for acute myocardial infarction and angina pectoris, may partly explain the decrease in the period effect for both sexes. Intracoronary thrombolysis was introduced in the late 1970s, percutaneous transluminal coronary angioplasty in the early 1980s, coronary stent implants in the early 1990s, and primary percutaneous coronary intervention (PCI) in the late 1990s.

The cohort effect for both sexes followed a downward trend between 1890 and 1910, increased and then peaked in 1930 for men and in 1925 for women, and then decreased again for both sexes. The temporary increase in the cohort effect might be partly due to the effects of World War I (1914–1918), World War II (1939–1945), and the subsequent period of classical Stalinism (1949–1953). Additionally, those born between 1910 and 1930 lived through World War II to the Stalinist era before the Hungarian Revolution of 1956 (and the subsequent years of retributions) as juveniles or young adults. Following World War II, living conditions were difficult, with loss of territory, railway networks, factories, livestock, and foreign trade, as well as inflation and rationing of food. These conditions might have therefore contributed to the increase in the cohort effect on IHD.

The effects of cohort and period differed between the capital and non-capital counties. The cohort effect for both sexes was larger in the capital for birth years between 1890 and 1925. During World War II, the primary military targets for bombings were concentrated in the capital, Budapest. In addition, the Battle of Budapest between the 24th of December 1944 and the 13th of February 1945 led to destruction and many casualties in the capital, deepening the difficulties in the subsequent years.^21^ These events might have contributed to the plateauing of the cohort effect for the capital between 1910 and 1925 for men, and between 1900 and 1925 for women. In non-capital counties, where agriculture was the primary industry, the situation was different from the capital. In spite of the loss of livestock and equipment, the land reform in 1945 gave private properties to Hungarian peasants in order to increase food production.^22^ The subsequent relatively better living conditions in non-capital counties may partly explain the decrease in the cohort effect for both sexes between 1890 and 1910. However, from 1948, forced collectivization (the elimination of private peasant farms and the establishment of agricultural cooperatives) and forced industrialization led to environmental pollution and decline in quality of life, especially among young adults in non-capital counties, possibly contributing to the increase in cohort effect between 1910 and 1925 for men and women in non-capital counties.^22^

After the birth cohort of 1925, the cohort effect became smaller in the capital than in non-capital counties, as did the period effect for both sexes. These regional differences can be explained partly by differences in the prevalence of obesity and lifestyles between the capital and non-capital counties. In 2002, the prevalence of obesity (body mass index ≥30) was approximately 1.5 times higher for individuals living in small towns and villages than for individuals living in the capital.^8^ The higher prevalence of obesity in non-capital counties might be due to consumption of traditional Hungarian foods with high calorie and fat content, as well as less participation in sport activities (29.6% in the capital and 19.7% in small towns and villages).^8^^–^^10^ Another possible explanation is the different socioeconomic statuses between the capital and non-capital counties. According to the 2005 Hungarian micro-census, the proportions of primary, secondary, and higher educational qualifications among the employed population aged 40 to 84 years were 10.6%, 52.3%, and 37.0%, respectively, in the capital; respective rates in non-capital counties were 19.0%, 62.7%, and 18.3%.^12^ Proportions according to type of occupation also differed. Skilled workers for agriculture, forestry, craft, and related trades represented 18.2% of employees in the capital and 36.0% of employees in non-capital counties, while leaders and other intellectuals represented 62.8% and 40.5%, respectively.^12^ The unemployment rate was 5.4% in the capital and 12.2% in non-capital counties.^12^

The accessibility of health services also differed between the capital and non-capital counties during the period examined. By the end of the 1990s, five intervention centres in the capital started to perform primary PCI, while only four out of the 19 non-capital counties had a primary PCI centre at that time. By the end of 2010, only an additional seven non-capital counties had a primary PCI centre. In addition, between 2000 and 2006, the number of hospital beds in use per 1000 people and the number of annual working hours of physicians per 100 people were 84% and 115% larger, respectively, in the capital than the corresponding figures for the non-capital counties.^12^

The sudden dip in IHD mortality rates for both sexes in 1979 may be due to the change in coding rules in 1979 (from ICD-8 to ICD-9), while the sudden increase in IHD mortality rates in 2005 may be due to the change in the method of data processing from manual coding to automated processing.^12^ Because of the lack of long-term age-specific or region-specific data on smoking habits and alcohol consumption, it is hard to relate these risk factors to IHD mortality. To get a deeper understanding of IHD trends in Hungary, further research on IHD incidence and case-fatality rates for different age groups is needed.

In conclusion, our study showed increasing health disparity associated with IHD deaths between the capital and non-capital counties in Hungary, as well as the start of an overall decrease in IHD mortality rates in the 1990s. These trends may be partly attributable to geographical differences in coronary risk factors, socio-economic status, and accessibility of health services. Our results indicate a need for comprehensive programs for IHD prevention in Hungary, such as health education, risk factor screening, referral to physicians, and medical treatment of high-risk individuals, especially for individuals from non-capital counties.

## ONLINE ONLY MATERIALS

eFigure 1. Sex- and age-specific mortality rates of ischaemic heart disease in Hungary from 1970 to 2009. Rates are based on 5-year age groups for ages 40 to 84 years. Expected mortality rates calculated with the APC model (lines) are plotted together with the reported mortality rates (markers).

eFigure 2. Age-specific mortality rates of ischaemic heart disease for men from the capital and non-capital counties of Hungary from 1970 to 2009. Rates are based on 5-year age groups for ages 40 to 84 years. Expected mortality rates calculated with the APC model (lines) are plotted together with the reported mortality rates (markers).

eFigure 3. Age-specific mortality rates of ischaemic heart disease for women from the capital and non-capital counties of Hungary from 1970 to 2009. Rates are based on 5-year age groups for ages 40 to 84 years. Expected mortality rates calculated with the APC model (lines) are plotted together with the reported mortality rates (markers).

eFigure 4. Cigarette consumption in Hungary from 1970 to 2000.

eFigure 5. Trends in cigarette smoking prevalence in Hungary.

eFigure 6. Alcohol consumption in Hungary from 1970 to 2010.

Abstract in Japanese.
