# Supplementary material for: Health Disparities in Ischaemic Heart Disease Mortality in Hungary From 1970 to 2010: An Age-Period-Cohort Analysis
Source: J Epidemiol. 2015 Jun 5;25(6):399–406. doi: 10.2188/jea.JE20140122 (PMC4444493; doi:10.2188/jea.JE20140122)
Supplement: eFigure 3. [file je-25-399-s003.pdf]

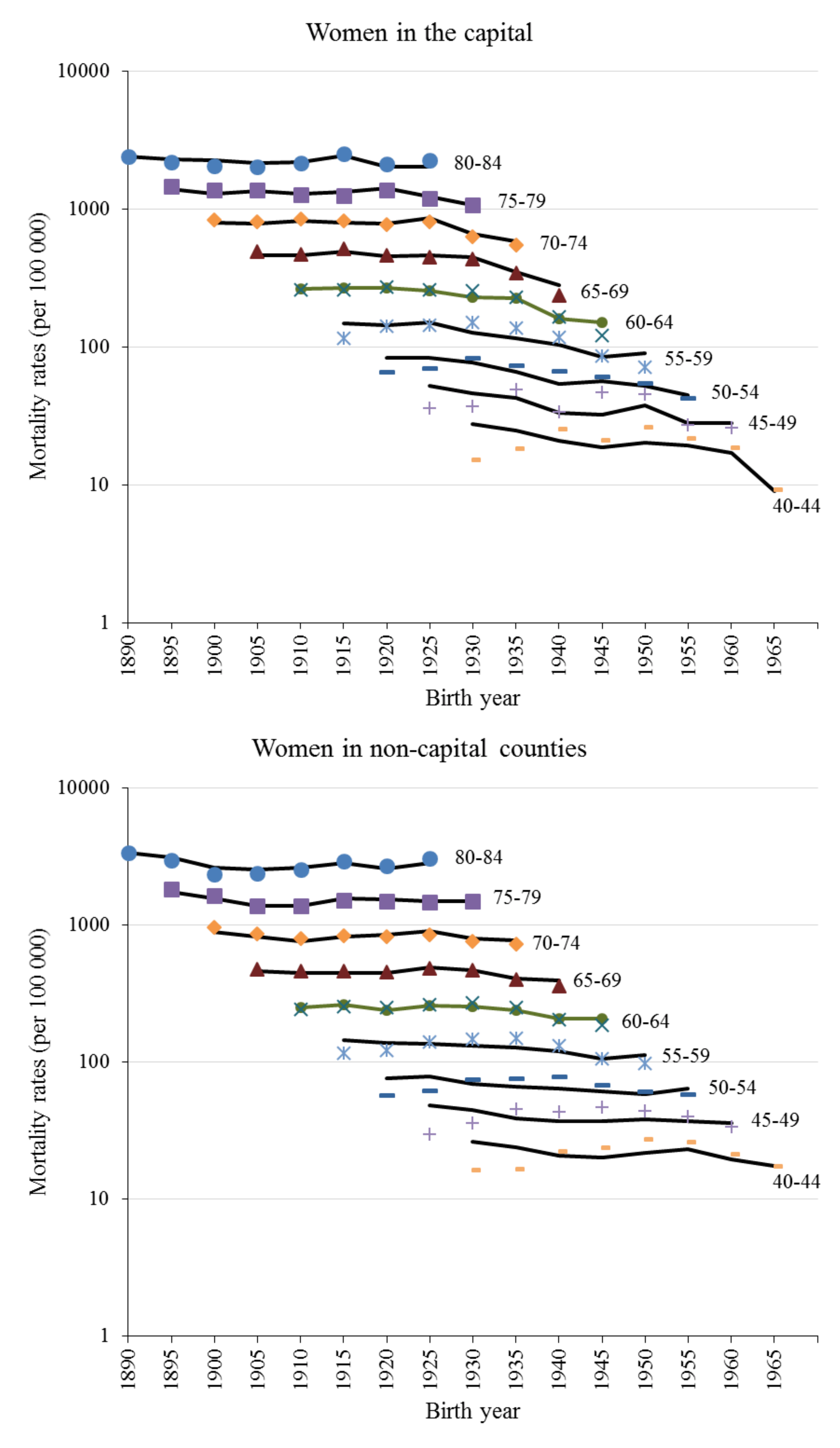

eFigure 3. Age-specific mortality rates of ischaemic heart disease for women from the capital and from non-capital counties of Hungary from 1970 to 2009. Rates are based on 5-year age groups for ages 40 to 84 years. Expected mortality rates calculated with the APC model (lines) are plotted together with the reported mortality rates (markers).
