# Supplementary material for: Health Disparities in Ischaemic Heart Disease Mortality in Hungary From 1970 to 2010: An Age-Period-Cohort Analysis
Source: J Epidemiol. 2015 Jun 5;25(6):399–406. doi: 10.2188/jea.JE20140122 (PMC4444493; doi:10.2188/jea.JE20140122)
Supplement: eFigure 4. [file je-25-399-s005.pdf]

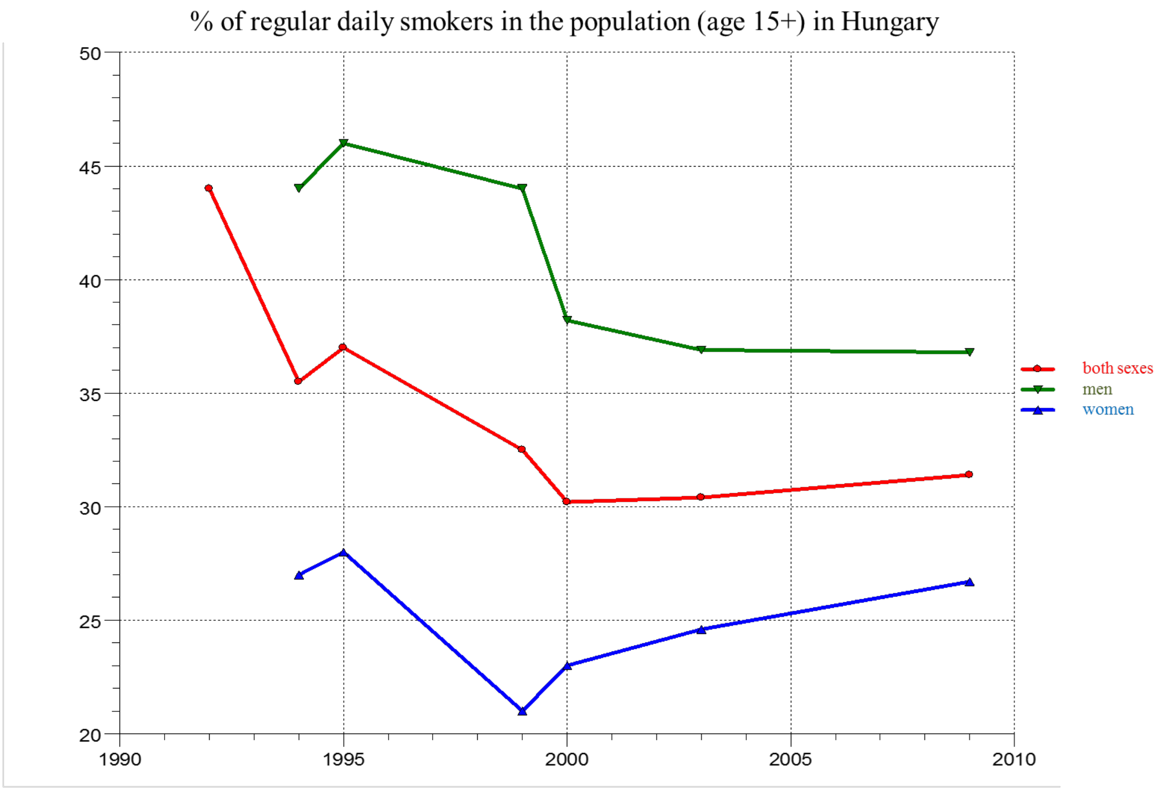

Source : WHO/Europe, European HFA Database, April 2014

eFigure 5. Trends in cigarette smoking prevalence in Hungary.
