# Supplementary figures and images for: Health Disparities in Ischaemic Heart Disease Mortality in Hungary From 1970 to 2010: An Age-Period-Cohort Analysis
Source: J Epidemiol. 2015 Jun 5;25(6):399–406. doi: 10.2188/jea.JE20140122 (PMC4444493; doi:10.2188/jea.JE20140122)

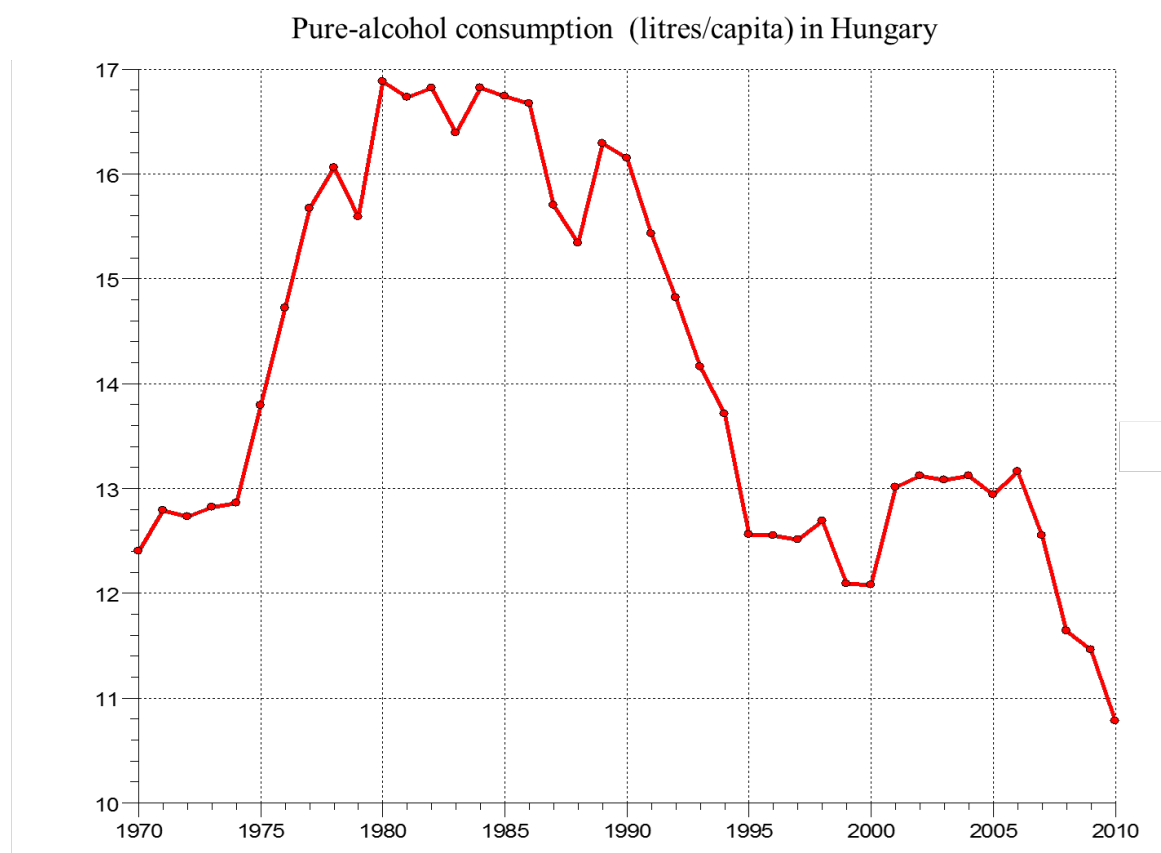

Source : WHO/Europe, European HFA Database, April 2014

**eFigure 6.** Alcohol consumption in Hungary from 1970 to 2010.

Supplement: eFigure 6. [file je-25-399-s006.pdf]
