# Supplementary material for: Health Disparities in Ischaemic Heart Disease Mortality in Hungary From 1970 to 2010: An Age-Period-Cohort Analysis
Source: J Epidemiol. 2015 Jun 5;25(6):399–406. doi: 10.2188/jea.JE20140122 (PMC4444493; doi:10.2188/jea.JE20140122)
Supplement: Abstract in Japanese. [file je-25-399-s007.pdf]

1970 年から 2010 年のハンガリーにおける虚血性心疾患死亡率の健康格差：age-period-cohort 分析

ゲロー　クリスティーナ[1]、エシャク　イーハブ　サラホ[1]、馬 恩博[2]、高橋　秀人[2]、野田博之[3]、磯　博康[1]

[1]大阪大学大学院医学系研究科社会医学講座公衆衛生学
[2]筑波大学医学医療系臨床試験・臨床疫学
[3]厚生労働省健康局がん対策・健康増進課、

目的
本研究の目的は、ハンガリーにおける主要な死因のうち、虚血性心疾患（IHD）死亡率の長期的な傾向を検討することにある。本研究では、ハンガリー全土ならびに首都（ブダペスト）と他郡の比較について、IHD 死亡率の APC(age-period-cohort)分析を行った。

方法
IHD による死亡と人口センサスのデータは、ハンガリー中央統計局から得た。APC 分析は、40~84 歳を対象として 5 年齢ごとの 9 クラスとし、1970~2009 年までの死亡を 8 期間に分けて、1886~1969 年までの 16 コホートを構築した。

結果
年齢調整 IHD 死亡率は、男性では 1970 年から 1993 年、女性では 1980 年から 1990 年までは上昇傾向であったが、その後は男女ともに減少した。さらに、男性では 1991 年、女性では 1970 年以降は、首都の IHD 死亡率は他郡よりも低く、その差が 1990 年以降は増大した。年齢効果は、男性よりも女性のほうが大きく影響した。男性の時代効果は、1972 年～1982 年は増加し、その後は減少した一方で、女性は 1972 年～2007 年の間は一貫して減少した。首都での時代効果の減少は、男女ともに他郡よりも低かった。男女ともに
コホート効果は 1890 年～1965 年の出生者で減少し、また、首都におけるコホート効果は、他郡よりも急峻に減少した。

結論
ハンガリーにおいて、特に首都以外の郡における IHD 防止のためのプログラムの必要性が示された。

キーワード：虚血性心疾患、死亡率、年齢·時代·コホート分析、ハンガリー
